# Association between Vitamin Intake and Chronic Kidney Disease According to a Variant Located Upstream of the *PTGS1* Gene: A Cross-Sectional Analysis of Shika Study

**DOI:** 10.3390/nu14102082

**Published:** 2022-05-16

**Authors:** Kim-Oanh Pham, Akinori Hara, Hiromasa Tsujiguchi, Keita Suzuki, Fumihiko Suzuki, Sakae Miyagi, Takayuki Kannon, Takehiro Sato, Kazuyoshi Hosomichi, Hirohito Tsuboi, Thao Thi Thu Nguyen, Yukari Shimizu, Yasuhiro Kambayashi, Masaharu Nakamura, Chie Takazawa, Haruki Nakamura, Toshio Hamagishi, Aki Shibata, Tadashi Konoshita, Atsushi Tajima, Hiroyuki Nakamura

**Affiliations:** 1 Information Management Department, Asia Center for Air Pollution Research, Niigata City 950-2144, Japan; 2 Department of Hygiene and Public Health, Faculty of Medicine, Institute of Medical, Pharmaceutical and Health Sciences, Kanazawa University, Kanazawa City 920-8640, Japan; hara-akinori@med.kanazawa-u.ac.jp (A.H.); t-hiromasa@med.kanazawa-u.ac.jp (H.T.); keitasuzuk@stu.kanazawa-u.ac.jp (K.S.); f-suzuki@den.ohu-u.ac.jp (F.S.); m.nakamura.83-7-7@r.vodafone.ne.jp (M.N.); takazawa@staff.kanazawa-u.ac.jp (C.T.); haruki_nakamura_kanazawa@yahoo.co.jp (H.N.); hamagisi@chubu-gu.ac.jp (T.H.); akintoki1116@gmail.com (A.S.); hnakamu@staff.kanazawa-u.ac.jp (H.N.); 3 Community Medicine Support Dentistry, Faculty of Dentist, Ohu University Hospital, Koriyama 963-8611, Japan; 4 Innovative Clinical Research Center, Takaramachi Campus, Kanazawa University, Kanazawa City 920-8640, Japan; smiyagi@staff.kanazawa-u.ac.jp; 5 Department of Bioinformatics and Genomics, Faculty of Medicine, Institute of Medical, Pharmaceutical and Health Sciences, Kanazawa University, Kanazawa City 920-8640, Japan; kannon@med.kanazawa-u.ac.jp (T.K.); tsato@med.kanazawa-u.ac.jp (T.S.); khosomic@med.kanazawa-u.ac.jp (K.H.); atajima@med.kanazawa-u.ac.jp (A.T.); 6 Institute of Medical, Pharmaceutical and Health Sciences, Kakuma Campus, Kanazawa University, Kanazawa City 920-1192, Japan; tsuboih@p.kanazawa-u.ac.jp; 7 Department of Epidemiology, Faculty of Public Health, Haiphong University of Medicine and Pharmacy, Hai Phong 180000, Vietnam; nttthao@hpmu.edu.vn; 8 Department of Nursing, Faculty of Health Sciences, Komatsu University, Komatsu City 923-0961, Japan; h_zu@me.com; 9 Department of Public Health, Faculty of Veterinary Medicine, Okayama University of Science, Imabari 794-8555, Japan; y-kambayashi@vet.ous.ac.jp; 10 Third Department of Internal Medicine, Faculty of Medical Sciences, University of Fukui, Tsuruga 914-0055, Japan; konosita@u-fukui.ac.jp

**Keywords:** chronic kidney disease, eGFR, nutritional vitamins, *PTGS1*, precision nutrition

## Abstract

Chronic kidney disease (CKD) patients have been advised to take vitamins; however, the effects have been controversial. The individual differences in developing CKD might involve genetic variants of inflammation, including variant rs883484 located upstream of the prostaglandin-endoperoxide synthase 1 (*PTGS1*) gene. We aimed to identify whether the 12 dietary vitamin intake interacts with genotypes of the rs883484 on developing CKD. The population-based, cross-sectional study had 684 Japanese participants (≥40 years old). The study used a validated, brief, self-administered diet history questionnaire to estimate the intake of the dietary vitamins. CKD was defined as estimated glomerular filtration < 60 mL/min/1.73 m^2^. The study participants had an average age of 62.1 ± 10.8 years with 15.4% minor homozygotes of rs883484, and 114 subjects had CKD. In the fully adjusted model, the higher intake of vitamins, namely niacin (odds ratio (OR) = 0.74, 95% confidence interval (CI): 0.57–0.96, *p* = 0.024), α-tocopherol (OR = 0.49, 95% CI: 0.26–0.95, *p* = 0.034), and vitamin C (OR = 0.97, 95% CI: 0.95–1.00, *p* = 0.037), was independently associated with lower CKD tendency in the minor homozygotes of rs883484. The results suggested the importance of dietary vitamin intake in the prevention of CKD in middle-aged to older-aged Japanese with minor homozygous of rs883484 gene variant.

## 1. Introduction

Chronic kidney disease (CKD) is a global burden, with approximately 10% of adults affected worldwide [1]. The disease is a progressive chronic condition, which can escalate to severe damage to organs, necessitating either dialysis or organ transplant to preserve life. Significantly, the aging population, such as the Japanese with degrading renal functions, is vulnerable to CKD [2]. Recently, precision nutrition has helped design beneficial prevention approaches for individuals based on their genetic profile [3,4]. Vitamin and genetic factors are both known to contribute to susceptibility to CKD [5,6]; however, there is not much information about the interaction between vitamin intake and gene variants on CKD development.

*PTGS1* (prostaglandin-endoperoxide synthase 1) gene, involved in the inflammatory response, expresses the enzyme catalyzing the conversion of arachidonic acid to prostaglandin (PG). It encodes cyclooxygenase 1 (COX-1) and peroxidase proteins and regulates angiogenesis in endothelial cells. *PTGS1* is one of the genes regulating blood pressure and arachidonic acid metabolism; it is one of the potential gatekeeper genes in programmed hypertension [7]. Thus, most of the studies about the expression of the *PTSG1* gene are on cardiovascular diseases [8,9].

A series of studies in the Japanese population identified rs883484 located upstream of the *PTGS1* gene as one of the genetic variants associated with CKD in individuals with metabolic syndrome [10], hypertension [11], and the general population [12]. The frequency of rs883484 polymorphism (CC:CT:TT) was 38:48:14 in general Japanese (> 60 years) [12] and 68:28:3 in Sweden males (>50 years old) [8]. The results suggested that *PTGS1* might also be susceptible to CKD among the Japanese.

The dietary vitamins among CKD patients are essential in controlling the progression rate of kidney failure, reducing uremic toxicity and proteinuria, and lowering the risk of kidney disease-related secondary complications [13,14]. Vitamins (water-soluble and fat-soluble vitamins) are recommended for CKD patients to control oxidant and inflammation levels [14]. CKD patients are inclined to vitamin deficiency due to gastrointestinal symptoms, dietary restriction, multi pharmacological prescription, or dialysis [15]. Moreover, the risk of vitamin malnutrition rises with age [16]. Even though the vitamins are good for CKD patients, the results are conflicting [17], in which genetic factors might be involved. Therefore, the relationship between vitamins and gene variants on CKD needed investigation. 

Disease prevention is an important strategy to control the burden of CKD, and the identification of genetic and nutritional factors is crucial for predicting risk and plausible intervention. The interaction between vitamin intake and genetic factors underlying the predisposition of CKD in individuals has remained unknown. Thus, the present study was to determine the effect of vitamin intake on CKD according to the rs883484 variant located upstream of the *PTGS1* gene in a middle-aged to older-aged population in Shika Town, Japan. 

## 2. Materials and Methods

### 2.1. Study Design and Population

This cross-sectional study utilized the data from Shika Cohort Study conducted from 2013 to 2019 in Shika Town, a rural area in Japan. Besides obtaining information from the self-administered questionnaires, genotypes of genome-wide single-nucleotide polymorphisms (SNPs) and daily salt intake were acquired from the comprehensive medical check-ups. 

**Figure 1:**
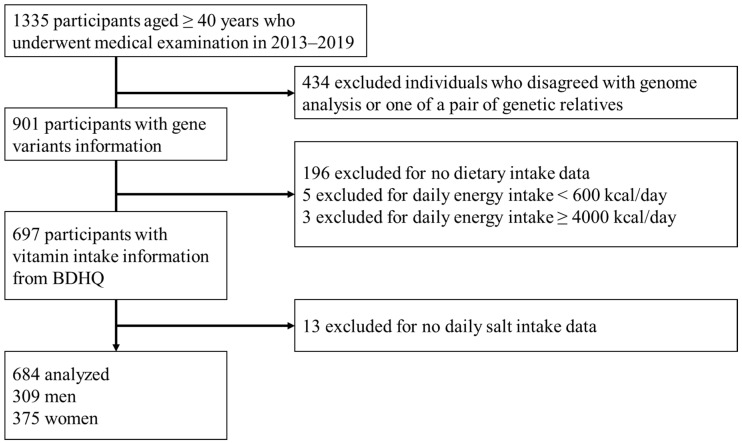
Flow chart showing the study enrolment procedure.

The number of eligible participants determined the sample size. There were 1335 voluntary participants from 40 years old with medical records (Figure 1). Excluded were individuals who disagreed with genome analysis or one of a pair of genetic relatives (*n* = 434), those without dietary intake information (*n* = 196) and daily salt intake (*n* = 13), and those with daily energy intake of <600 kcal/day or >4000 kcal/day (*n* = 8); thus, the present study included 684 participants.

### 2.2. Genotyping of rs883484

Genomic DNA was extracted from blood samples utilizing the QIAamp DNA Blood Maxi Kit (Qiagen, Hilden, Germany) corresponding to the manufacturer’s instructions or entrusting to a specialized company in clinical laboratory testing (SRL, Inc., Tokyo, Japan). Genome-wide SNP genotyping was executed using the Japonica Array v2 [18] (TOSHIBA Co., Ltd., Tokyo, Japan). Quality control (QC) procedures for the obtained genome-wide SNP genotype data have been described elsewhere [19]. In brief, SNP genotype data and participants were filtered according to gender identity between karyotypes and the questionnaire, call rates, the Hardy–Weinberg equilibrium test, inbreeding coefficient, cryptic relatedness, and population structure. Genotypes of rs883484 for 901 unrelated subjects (based on genome-wide $\hat{\pi }$ values) passing the QC were extracted from the array data. In the QC step, the call rate for the SNP was 100%, and a departure from the Hardy–Weinberg equilibrium was not observed (*p* = 0.9082).

### 2.3. Assessment of Chronic Kidney Disease

To evaluate the kidney function, estimated glomerular filtration rate (eGFR) was calculated following the simplified equation proposed by Japanese Society of Nephrology and described in the Modification of Diet in Renal Disease Study: eGFR (mL/min/1.73 m^2^) = 194 × [age (years)]^−0.287^ × [serum creatinine (mg/dL)]^−1.094^ × [0.739 if female] [20]. The enzymatic method was used to quantify the serum creatinine concentrations. In this study, the criteria to define CKD was that the eGFR is less than 60 mL/min/1.73 m^2^ [21]. The eGFR is an estimated value that might not reflect the exact GFR, and the cutoff point to determine CKD might cause bias in the analysis.

### 2.4. Vitamin Intake Assessment

The intake of water-soluble vitamins (vitamin C, B_1_, B_2_, niacin, pantothenic acid, B_6_, folate, and B_12_) and fat-soluble vitamins (α-tocopherol, vitamin D, K, and retinol) was evaluated by the Japanese version of the brief self-administered dietary history questionnaire (BDHQ). These vitamins are recommended for CKD patients to improve their renal condition. BDHQ assembles information on the average intake of 58 types of food and beverage during the previous month to estimate the habitual intake of nutrients. The vitamin intake was estimated using a computer algorithm for the BDHQ based on the Standard Tables of Food Composition in Japan [22]. To avoid bias from excessive intake of nutritional supplements, mineral or vitamin intake from supplements was not included in the calculation. The Japanese version of BDHQ has been validated in previous studies [22,23]. The nutritional intake adjusted for energy using the density method was performed instead of the crude values of the dietary record. 

### 2.5. Other Variables

Blood pressure (BP) was measured twice consecutively at rest in a sitting position, and averages were used as BP data. Hypertension was set as systolic blood pressure (SBP) of ≥140 mm Hg diastolic blood pressure (DBP) of ≥90 mm Hg. Glycated hemoglobin (HbA_1c_) and fasting plasma glucose (FPG) were measured during the medical examination. Body mass index (BMI) was calculated from weight and height in the follow-up appointments and expressed as kg/m^2^. Diabetes was described as HbA_1c_ of ≥6.5% or FPG ≥ 126 mg/dL, which is the cutoff value used by the Japan Society to diagnose diabetes mellitus, or previously diagnosed as diabetes [24]. The daily salt intake was estimated from the daily urinary sodium excretion by the Japanese Society of Hypertension Guidelines 2014 [25].

Age (set at the time of measurement), sex, smoking status, alcohol consumption, and exercise frequency were evaluated using self-administered questionnaires. Habitual alcohol consumption was determined as drinking more than one glass of sake (22 g ethanol) per day three times a week or more [26]. The decent frequency of exercise was estimated as follows: participants exercised more than 30 min at least twice a week during the preceding year or performed tasks such as walking, cleaning, and carrying baggage for more than one hour per day [26]. The categorical variables were presented as binary: sex (0 = woman, 1 = man), hypertension prevalence (0 = no hypertension, 1 = hypertension), diabetes prevalence (0 = no diabetes, 1 = diabetes), smoking status (0 = non-smoker or ex-smoker, 1 = current smoker), drinking habit (0 = non-drinker, 1 = drinker), and exercise frequency (0 = non-exercise, 1 = frequent exercise).

### 2.6. Statistical Analysis

Descriptive characteristics at baseline were compared according to the CKD status and rs883484 genotypes. The CKD status is based on the presence or absence of eGFR < 60 mL/min/1.73 m^2^ (0 = non-CKD, 1 = CKD). The genotypes were divided into major homozygous and heterozygous (CC + CT) and minor homozygous (TT). The independent-sample Student’s *t*-test was used to compare the continuous data between subjects with different CKD statuses and rs883484 genotypes, and the chi-square test compared categorical data. The interaction between vitamin intake and rs883484 genotypes on CKD was performed using a two-way Analysis of Covariance (ANCOVA). A multiple logistic regression analysis was utilized to examine the association between vitamin intake and CKD status according to the rs883484 genotypes after adjustments for the following independent factors: age, sex, BMI, hypertension, diabetes, drinking habits, exercise habits, smoking habits, and the daily salt intake. 

All statistical analyses in the present study used the IBM SPSS Statistics version 25.0 (IBM Corp., Tokyo, Japan). Odds ratios (OR), *p*-values, and 95% confidence intervals (CI) were calculated. The results with a two-sided *p*-value < 0.05 were considered to be significant.

## 3. Results

### 3.1. Participant Characteristics According to CKD Status

The studied participants were 684 after the screening process, which is shown in Figure 1. The sample size produced the effect size of 0.11 with ten covariates in two groups, with an 80% power and 5% significance. Participant characteristics, lifestyle habits, frequency distributions of rs883484 genotypes, and nutrient intake are summarized according to CKD status in Table 1. Among 684 participants (62.1 (SD, 10.8) years old), 54.8% (*n* = 375) were women, and 114 (16.6%) had CKD. The minor homozygous of rs883484 was 15.4%, and the major homozygous and heterozygous group was 84.6% of the studied population. Age (*p* = 0.000), BMI (*p* = 0.023), the prevalence of hypertensive subjects (*p* = 0.009), and SBP (*p* = 0.001) were significantly higher in the CKD group than those in the non-CKD group. There were more females (*p* = 0.018) and current smokers (*p* = 0.002) and higher daily salt intake (*p* = 0.001) in the non-CKD group than in the CKD group, while the vitamin C intake (*p* = 0.037) was significantly lower in the non-CKD group than that in the CKD group. The ratio of frequently exercising people (*p* = 0.088) in the CKD group was borderline significantly higher than that in the non-CKD group. Diabetes individuals and lipid profiles (triglyceride and total cholesterol) were not significantly different between groups. Except for vitamin C, there was no significant difference in the consumption of vitamins intake between groups.

### 3.2. Participant Characteristics According to rs883484 Genotypes and CKD Status

In Table 2, there were two groups according to gene rs883484 genotypes: the major homozygous and heterozygous (CC + CT) group versus the minor homozygous (TT) group, which were further divided by CKD status. Overall, 16.9% and 15.2% of participants had CKD in CC + CT and TT groups, respectively. Age significantly affected CKD in both genotype groups. In both genotype groups, the daily salt intake was lower in the CKD group than in the non-CKD group: *p* = 0.003 (CC + CT group) and *p* = 0.060 (TT group). In the minor homozygous group, the intake of the vitamins was lower in the CKD group, with significant differences in niacin (*p* = 0.027) and α-tocopherol (*p* < 0.050). Diabetes preferences and lipid profiles were not significantly different between groups.

**Figure 2:**
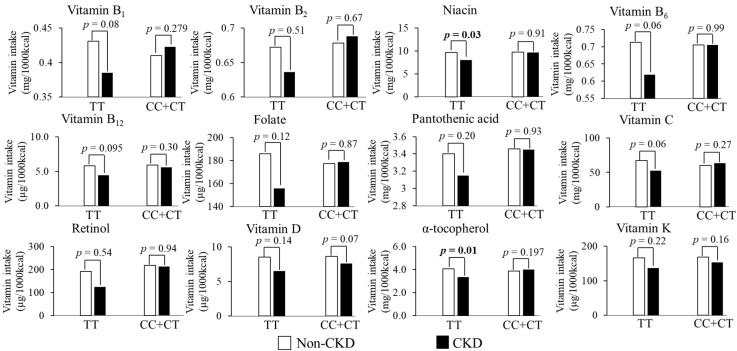
Interaction between vitamin intake and rs883484 genotype in the recessive model on CKD by two-way ANCOVA with adjustment for age, sex, BMI, hypertension, diabetes, smoking status, drinking, and exercise habits. *p*-Values were from the Bonferroni test.

In CC + CT group, age (*p* < 0.001), BMI (*p* = 0.002), SBP (*p* < 0.001), and prevalence of hypertension (*p* = 0.006) was significantly higher in CKD group. There were more females (*p* = 0.009) and current smokers (*p* < 0.001) in the non-CKD group than in the CKD group. In contrast to the TT group, there was a significant difference in vitamin C intake (*p* = 0.012) and a marginally significant difference in α-tocopherol intake (*p* = 0.067) between the groups: the CKD group consumed more vitamin C and α-tocopherol than the non-CKD group. There was a significant interaction between CKD groups and rs883484 genotypes on the intake of vitamins (*p* = 0.028 for vitamin C, *p* = 0.041 for vitamin B_1_, *p* = 0.046 for niacin, *p* = 0.005 for α-tocopherol) (Table 3). Niacin (*p* = 0.03) and α-tocopherol (*p* = 0.01) showed significant results, while vitamin B_1_, B_6_, B_12_, and vitamin C had borderline significance on Bonferroni analysis (Figure 2). In the CC + CT group, the intake of vitamins did not affect the CKD, whereas in the TT group, the higher intake of vitamins resulted in lower CKD outcomes. The result suggests a relationship between the intake of vitamins and CKD according to the gene variant. From descriptive results in Table 1 and Table 2, age, sex, BMI, hypertension, smoking, and daily salt intake strongly influenced CKD outcomes. Diabetes, drinking habits, and exercise were strong covariates [1,3,4,26]. All 684 subjects had no missing data in the confounder and main effect variables.

### 3.3. Relationship between Antioxidant Vitamins Intake and CKD According to Gene Variants

The effect of the consumption of antioxidant vitamins on CKD stratified by rs883484 genotypes is presented in Table 4. The fully adjusted model included age, sex, BMI, hypertension, diabetes, and lifestyle habits, including drinking, smoking, exercise, and daily salt intake. The results showed that high consumption of niacin (OR = 0.739, 95% CI: 0.569–0.961, *p* = 0.024), α-tocopherol (OR = 0.492, 95% CI: 0.256–0.947, *p* = 0.034), vitamin C (OR = 0.971, 95% CI: 0.945–0.998, *p* = 0.037), folate (OR = 0.987, 95% CI: 0.997–1.003, *p* = 0.063), vitamin B_6_ (OR = 0.037, 95% CI: 0.001–1.131, *p* = 0.059), pantothenic acid (OR = 0.407, 95% CI: 0.153–1.078, *p* = 0.070), and vitamin B_1_ (OR = 0.003, 95% CI: 0.000–1.773, *p* = 0.075) inversely correlated with CKD in TT group after adjustment. In the CC + CT group, the association between vitamin intake and CKD was insignificant after adjustment.

## 4. Discussion

The present study discovered that a higher intake of water-soluble vitamins and fat-soluble vitamins, especially niacin, vitamin C, and α-tocopherol, was independently associated with lower CKD tendency in the Japanese population minor homozygote of rs883484 located upstream of *PTGS1* gene. In contrast, the participants with the C allele showed no significant effect of vitamins on CKD status. In addition, the association between the intake of fat-soluble vitamins (except for α-tocopherol), vitamin B_2_, and vitamin B_12_ and kidney function was not observed in both groups of rs883484 genotypes. Unexpectedly, the salt intake in the CKD group was lower than that in the non-CKD group. Hence, the effect of elevated blood pressure by consuming salt on CKD might be excluded.

The present results for vitamins intake are consistent with previous studies indicating the protective effect of dietary vitamins intake and CKD. Vitamin C, niacin, and α-tocopherol significantly affected CKD status. Moreover, the lower intake of vitamins B_1_, B_6_, pantothenic acid, and folate were borderline significantly associated with higher CKD prevalence. Niacin (nicotinic acid, vitamin B_3_) is the oldest drug to treat dyslipidemia [27,28] and hyperphosphatemia [29]. Niacin reduces serum phosphorus levels and oxidative stress, including inflammation and endothelial dysfunction, and improves the CKD outcome [30]. Vitamin C is vital for many biological functions and attenuates reactive oxygen species, and renal oxidative damage preserves hydroxylase and monooxygenase enzymes and endothelium and vascular function in renal injury patients [31]. However, vitamin C deficiency is common among CKD patients due to dialytic vitamin C clearance, high vitamin C food restriction, and escalated vitamin C catabolism in vivo from inflammation [14,15]. α-Tocopherol, which belonged to the vitamin E group, was inversely associated with CKD in middle-aged and older-aged Japanese women [26]. The intake of vitamins C and E alone or in combination lessened kidney function damage, renal injury, and arterial pressure in rats with salt-sensitive hypertension [32]. Even though vitamins have been reported to be protective against CKD because of their antioxidant and anti-inflammatory [14], the results were not statistically significant [27].

In the CC + CT group, the intake of vitamins did not alter the outcome of CKD, while in the TT group, it drastically changed the CKD status. Yoshida et al. showed that the rs883484 (C/T) located upstream of the *PTGS1* gene was associated with CKD in the general population [12] and individuals with metabolic syndrome [10] as well as hypertension [11] patients. The SNP is located in the 5′ flanking region [8], which contains the promoter and may contain enhancers or other protein binding sites. Polymorphisms in this region can induce changes in the regulation of transcription. Helmersson et al. displayed that the TT genotype of rs883484 was associated with the increased formation of PGF_2α_, a product of COX [8]. *PTGS1* gene encodes COX-1, which synthesizes PG and thromboxane (Tx), which leads to inflammatory damage to the kidney [33]; thus, the COX pathway might be the mechanism for developing CKD in the case of TT genotype for rs883484. The previous findings suggested that the minor homozygote (TT genotype) of rs883484 increased the *PTGS1* gene expression and the amount of COX-1, which worsened renal function. COX-1 is the primary target of nonsteroidal anti-inflammatory drugs (NSAIDs). Inhibition of PGs by pharmacological medicine enhances immunocompetence in animals and humans. Likewise, the supplement of vitamins has a similar effect on PGs. Vitamins C and E inhibited PGE_2_ production by human gingival fibroblasts and SCC-25 oral squamous carcinoma cells [34]. Plasma PGE_2_ concentrations decreased in men and women after 14 days of using vitamin C-rich vegetable soup [35]. Niacin regulates PGF_2α_ [36], PGE_2_ [37], and PGD_2_ [38] through COX-1 and COX-2. Because of the enhanced expression of COX-1, individuals with TT genotype of rs883484 might become more sensitive to the antioxidant and anti-inflammatory effects of vitamins. Thus, the TT genotype of rs883484 seemed to be more susceptible to dietary intake of vitamins against kidney failure.

This study has several limitations. First, this cross-sectional study cannot establish a causal relationship, which means we cannot determine that the dietary intake of antioxidant vitamins can prevent CKD development in the general population with minor homozygotes of rs883484. Second, the estimated GFR was used instead of directly measured GFR to define CKD. Third, the self-administered dietary history questionnaire (BDHQ) cannot accurately determine the nutrition intake due to a limited number of food and beverage items. Moreover, there is a lack of objective markers of daily vitamin intake and energy intake.

## 5. Conclusions

In conclusion, TT allele carriers of the rs883484 variant, occupying 15.4% of the studied population, had a high risk of CKD. However, dietary vitamin intake is inversely associated with CKD in minor homozygotes of rs883484. A diet rich in vitamins would benefit those with a minor homozygous rs883484 variant. Major homozygote and heterozygote of rs883484 did not respond to vitamin intake to protect against CKD. The current study was conducted on the middle-aged to older-aged Japanese general population; therefore, its finding has practical meaning to generalize. Further primary and longitudinal studies using objective markers of vitamin intake are needed to examine the underlying mechanism preventing CKD development.

## Figures and Tables

**Table 1:** Participant characteristics in different CKD statuses.

	Total (*n* = 684)	non-CKD (*n* = 570)	CKD (*n* = 114)	*p*-Value *^6^
	Mean (SD)	Mean (SD)	Mean (SD)	
Characteristics				
Age (years)	62.1 (10.8)	60.7 (10.5)	68.9 (9.6)	**0.000**
Female, *n* (%)	375 (54.8)	324 (56.8)	51 (44.7)	**0.018**
BMI *^1^ (kg/m^2^)	23.2 (3.2)	23.1 (3.1)	23.9 (3.8)	**0.023**
SBP *^2^ (mmHg)	138.7 (19.4)	137.5 (19.1)	144.4 (20.0)	**0.001**
DBP *^3^ (mmHg)	80.1 (11.4)	80.1 (11.2)	80.2 (12.2)	0.900
Hypertension, *n* (%)	320 (46.8)	254 (44.6)	66 (57.9)	**0.009**
HbA1c *^4^ (%)	5.9 (0.7)	5.9 (0.7)	6.0 (0.5)	0.406
FPG *^5^ (mg/dL)	96.5 (18.0)	96.3 (18.2)	97.6 (16.9)	0.526
Diabetes, *n* (%)	95 (13.9)	76 (13.3)	19 (16.7)	0.347
Triglyceride (mg/dL)	118.6 (85.9)	117.4 (87.1)	124.6 (79.9)	0.411
Total cholesterol (mg/dL)	215.0 (34.5)	215.7 (34.9)	211.5 (32.1)	0.240
Lifestyle habit				
Current smoker, *n* (%)	130 (19.0)	120 (21.1)	10 (8.8)	**0.002**
Current drinker, *n* (%)	331 (48.4)	279 (48.9)	52 (45.6)	0.516
Frequent exercise, *n* (%)	407 (40.5)	331 (58.1)	76 (66.7)	0.088
Genotypes of rs883484				0.669
TT, *n* (%)	105 (15.4)	89 (15.6)	16 (14.0)	
CC + CT, *n* (%)	579 (84.6)	481 (84.4)	98 (86.0)	
Nutrition intake				
Vitamin B_1_ (mg/1000 kcal)	0.4 (0.1)	0.4 (0.1)	0.4 (0.1)	0.352
Vitamin B_2_ (mg/1000 kcal)	0.7 (0.2)	0.7 (0.2)	0.7 (0.2)	0.251
Niacin (mg/1000 kcal)	9.7 (2.8)	9.7 (2.8)	9.4 (3.2)	0.285
Vitamin B_6_ (mg/1000 kcal)	0.7 (0.2)	0.7 (0.2)	0.7 (0.2)	0.674
Vitamin B_12_ (μg/1000 kcal)	5.8 (3.1)	5.9 (3.1)	5.7 (3.1)	0.693
Folate (μg/1000 kcal)	178.2 (75.7)	176.9 (76.3)	184.7 (72.8)	0.313
Pantothenic acid (mg/1000 kcal)	3.4 (0.8)	3.4 (0.8)	3.5 (0.8)	0.569
Vitamin C (mg/1000 kcal)	61.1 (31.6)	59.9 (30.7)	67.4 (35.6)	**0.037**
Retinol (μg/1000 kcal)	210.9 (400.4)	213.4 (434.2)	198.8 (139.5)	0.722
Vitamin D (μg/1000 kcal)	8.4 (5.1)	8.5 (5.1)	8.2 (5.3)	0.649
α-tocopherol (mg/1000 kcal)	3.9 (1.1)	3.9 (1.1)	4.0 (1.2)	0.361
Vitamin K (μg/1000 kcal)	164.8 (92.8)	165.1 (93.5)	163.2 (89.9)	0.838
Daily salt intake (g/day)	9.5 (2.4)	9.7 (2.4)	8.8 (2.4)	**0.001**

*^1^ Body mass index; *^2^ Systolic blood pressure; *^3^ Diastolic blood pressure; *^4^ Glycated hemoglobin. *^5^ Fasting plasma glucose. *^6^ *t*-tests for continuous variables and chi-square tests for categorical variables; *p*-values of <0.05 are shown in bold. SD, standard deviation.

**Table 2:** Participant characteristics in different rs883484 genotypes and CKD statuses.

	CC + CT (*n* = 579)					TT (*n* = 105)				
	non-CKD (*n* = 481)		CKD (*n* = 98)		*p*-Value *	non-CKD (*n* = 89)		CKD (*n* = 16)		*p*-Value
	Mean (*n*)	SD (%)	Mean (*n*)	SD (%)		Mean (*n*)	SD (%)	Mean (*n*)	SD (%)	
Characteristics										
Age (years)	60.6	10.4	69.0	9.8	**0.000**	61.3	10.9	68.0	8.3	**0.020**
Sex, *n* (%) Female	271	56.3	41	41.8	**0.009**	53	59.6	10	62.5	0.825
BMI (kg/m^2^)	23.1	3.1	24.2	3.6	**0.002**	22.8	2.9	22.3	4.5	0.637
SBP (mmHg)	137.7	18.7	145.3	19.8	**0.000**	136.6	21.2	138.6	21.3	0.722
DBP (mmHg)	80.2	11.3	80.1	12.4	0.985	79.6	10.9	80.7	11.6	0.708
Hypertension	217	45.1	59	60.2	**0.006**	37.0	41.6	7.0	43.8	0.871
HbA1c (%)	5.9	0.7	6.0	0.6	0.471	5.9	0.6	5.9	0.4	0.685
FPG (mg/dL)	96.5	18.3	98.2	17.3	0.415	95.8	17.8	93.0	13.5	0.611
Diabetes	65	13.5	16	16.3	0.464	11	12.4	3	18.8	0.489
Triglyceride (mg/dL)	117.7	91.1	125.6	83.3	0.425	115.7	61.6	118.6	56.4	0.863
Total cholesterol (mg/dL)	215.7	35.3	211.9	33.5	0.339	215.6	32.7	208.8	22.1	0.421
Lifestyle habit										
Current smoker, *n* (%)	105.0	21.8	6.0	6.1	**0.000**	15.0	16.9	4.0	21.1	0.436
Current drinker, *n* (%)	236.0	49.1	47.0	48.0	0.842	43.0	48.3	5.0	4.8	0.207
Frequent exercise, *n* (%)	280.0	58.2	67.0	68.4	0.062	51.0	42.7	9.0	56.3	0.938
Nutrition intake										
Vitamin B_1_ (mg/1000 kcal)	0.4	0.1	0.4	0.1	0.124	0.4	0.1	0.4	0.1	0.255
Vitamin B_2_ (mg/1000 kcal)	0.7	0.2	0.7	0.2	0.207	0.7	0.2	0.7	0.3	0.902
Niacin (mg/1000 kcal)	9.7	2.8	9.6	3.2	0.750	9.7	2.6	8.1	2.8	**0.027**
Vitamin B_6_ (mg/1000 kcal)	0.7	0.2	0.7	0.2	0.308	0.7	0.2	0.6	0.2	0.191
Vitamin B_12_ (μg/1000 kcal)	5.9	3.1	5.9	3.2	0.935	5.8	3.0	4.7	2.3	0.179
Folate (μg/1000 kcal)	175.2	77.6	187.6	74.9	0.147	186.0	68.5	167.1	56.8	0.302
Pantothenic acid (mg/1000 kcal)	3.4	0.8	3.5	0.8	0.379	3.4	0.8	3.3	0.9	0.499
Vitamin C (mg/1000 kcal)	58.5	30.2	68.7	37.3	**0.012**	67.1	32.4	58.9	21.6	0.214
Retinol (μg/1000 kcal)	217.3	468.8	209.6	145.4	0.872	192.2	140.7	132.3	66.4	0.099
Vitamin D (μg/1000 kcal)	8.5	5.1	8.4	5.4	0.923	8.5	5.2	7.1	4.8	0.332
α-tocopherol (mg/1000 kcal)	3.8	1.1	4.1	1.2	0.067	4.1	1.1	3.5	1.4	**<0.05**
Vitamin K (μg/1000 kcal)	165.0	95.0	165.5	92.4	0.966	165.7	85.6	149.2	73.4	0.471
Daily salt intake (g/day)	9.6	2.5	8.8	2.5	**0.003**	9.9	2.1	8.8	2.1	0.060

* *t*-tests for continuous variables and chi-square tests for categorical variables; *p*-values of <0.05 are shown in bold.

**Table 3:** Main effect of CKD status and rs883484 genotypes and their interaction on vitamin intakes.

	*p*-Value for CKD Status *	*p*-Value for Genotypes *	*p*-Value for Interaction *
Vitamin B_1_	0.238	0.545	**0.041**
Vitamin B_2_	0.658	0.326	0.435
Niacin	**0.039**	**0.037**	**0.046**
Vitamin B_6_	0.092	0.152	0.085
Vitamin B_12_	0.054	0.166	0.256
Folate	0.174	0.486	0.133
Pantothenic acid	0.229	0.103	0.252
Vitamin C	0.192	0.620	**0.028**
Retinol	0.551	0.338	0.591
Vitamin D	**0.038**	0.413	0.504
α-tocopherol	0.073	0.133	**0.005**
Vitamin K	0.097	0.475	0.569

* Two-way Analysis of Covariance (ANCOVA) was performed with adjustment for age, sex, body mass index, hypertension, diabetes, smoking status, drinking, and exercise habits. *p*-Values of <0.05 are shown in bold.

**Table 4:** Association between antioxidant vitamins intake and CKD according to rs883484 genotypes.

		CC + CT		*p*-Value	TT		*p*-Value
		OR	95% CI		OR	95% CI	
Model 1	Water-soluble vitamins						
	Vitamin B_1_	7.668	0.743–79.106	0.087	0.005	0.000–1.384	0.065
	Vitamin B_2_	1.522	0.509–4.547	0.452	0.081	0.003–2.497	0.151
	Niacin	1.008	0.930–1.093	0.840	0.744	0.582–0.951	**0.018**
	Vitamin B_6_	1.344	0.404–4.476	0.630	0.037	0.002–0.845	**0.039**
	Vitamin B_12_	0.972	0.899–1.052	0.488	0.807	0.641–1.017	0.069
	Folate	1.001	0.998–1.004	0.613	0.988	0.975–1.000	**0.046**
	Pantothenic acid	1.073	0.789–1.459	0.652	0.418	0.174–1.003	0.051
	Vitamin C	1.006	0.998–1.013	0.134	0.975	0.975–0.951	**0.042**
	Fat-soluble vitamins						
	Retinol	1.000	0.999–1.001	0.955	0.991	0.982–1.000	0.059
	Vitamin D	0.97	0.924–1.018	0.215	0.906	0.789–1.040	0.159
	α-tocopherol	1.22	0.983–1.514	0.071	0.495	0.272–0.900	**0.021**
	Vitamin K	0.999	0.999–0.996	0.372	0.993	0.985–1.001	0.098
Model 2	Water-soluble vitamins						
	Vitamin B_1_	5.557	0.493–62.609	0.165	0.006	0.000–2.213	0.089
	Vitamin B_2_	1.447	0.458–4.573	0.529	0.092	0.003–2.917	0.176
	Niacin	1.014	0.934–1.101	0.737	0.764	0.592–0.986	**0.039**
	Vitamin B_6_	1.265	0.368–4.351	0.709	0.055	0.002–1.373	0.077
	Vitamin B_12_	0.98	0.905–1.062	0.621	0.831	0.831–1.059	0.135
	Folate	1.000	0.997–1.004	0.834	0.988	0.976–1.001	0.073
	Pantothenic acid	1.032	0.748–1.423	0.850	0.435	0.176–1.079	0.073
	Vitamin C	1.004	0.996–1.012	0.323	0.973	0.947–0.999	**0.046**
	Fat-soluble vitamins						
	Retinol	1.000	0.999–1.001	0.911	0.992	0.983–1.001	0.097
	Vitamin D	0.971	0.925–1.019	0.231	0.925	0.803–1.066	0.283
	α-tocopherol	1.179	0.946–1.470	0.144	0.512	0.276–0.952	**0.034**
	Vitamin K	0.998	0.996–1.001	0.258	0.994	0.985–1.002	0.147
Model 3	Water-soluble vitamins						
	Vitamin B_1_	4.445	0.378–52.317	0.236	0.003	0.000–1.773	0.075
	Vitamin B_2_	1.187	0.369–3.824	0.773	0.067	0.002–2.582	0.147
	Niacin	1.006	0.926–1.094	0.880	0.739	0.569–0.961	**0.024**
	Vitamin B_6_	1.199	0.343–4.186	0.777	0.037	0.001–1.131	0.059
	Vitamin B_12_	0.978	0.902–1.060	0.589	0.823	0.633–1.069	0.144
	Folate	1.000	0.974–1.001	0.960	0.987	0.997–1.003	0.063
	Pantothenic acid	0.981	0.707–1.361	0.909	0.407	0.153–1.078	0.070
	Vitamin C	1.003	0.995–1.011	0.428	0.971	0.945–0.998	**0.037**
	Fat-soluble vitamins						
	Retinol	1.000	0.999–1.001	0.880	0.992	0.982–1.002	0.126
	Vitamin D	0.972	0.925–1.021	0.253	0.924	0.792–1.079	0.317
	α-tocopherol	1.164	0.929–1.458	0.187	0.492	0.256–0.947	**0.034**
	Vitamin K	0.999	0.996–1.001	0.271	0.994	0.985–1.003	0.181

OR, odds ratios; CI, confidence interval. *p*-values of <0.05 are shown in bold. Model 1: age, sex, body mass index (BMI), hypertension, and diabetes; Model 2: age, sex, BMI, hypertension, diabetes, exercise, smoking, and drinking; Model 3: age, sex, BMI, hypertension, diabetes, exercise, smoking, drinking, and salt intake.

## Data Availability

The data that support the present study’s findings are available from the corresponding author upon reasonable request.
